# Nutritional status and dietary intake before hospital admission of pulmonary tuberculosis patients

**DOI:** 10.3934/publichealth.2023031

**Published:** 2023-05-23

**Authors:** Trong Hung Nguyen, Thi Hang Nga Nguyen, Hung Le Xuan, Phuong Thao Nguyen, Kim Cuong Nguyen, Tuyet Nhung Le Thi

**Affiliations:** 1 National Institute of Nutrition, Hanoi, Vietnam; 2 Dong Anh General Hospital, Hanoi, Vietnam; 3 Hanoi Medical University, Hanoi, Vietnam; 4 Hanoi Andrology and Fertility Hospital, Hanoi, Vietnam

**Keywords:** tuberculosis, Subjective Global Assessment (SGA), dietary, National Lung Hospital

## Abstract

Conducting research on nutritional status and dietary intake of pulmonary tuberculosis patients is essential for developing interventions in clinical nutrition practice and treatment during hospitalization, which can improve the quality of patients life. This cross-sectional descriptive study aimed to determine nutritional status and some related factors (such as geography, occupation, educational level, economic classification, etc.) of 221 patients with pulmonary tuberculosis who were examined and treated at the Respiratory Tuberculosis Department, National Lung Hospital in July 2019–May 2020. The results showed that the risk of undernutrition: According to BMI (Body Mass Index): 45.8% of patients were malnourished, 44.2% normal and 10.0% overweight/obese. According to MUAC (Mid-Upper Arm Circumference): 60.2% of patients were malnourished, 39.8% of patients were normal. According to SGA (Subjective Global Assessment): 57.9% of patients were at risk of undernutrition, of which 40.7% were at moderate risk of undernutrition and 17.2% risk of severe undernutrition. Classification of nutritional status according to serum albumin index: 50% of patients were malnourished, the rate of undernutrition of mild, moderate and severe levels was 28.9%, 17.9% and 3.2%, respectively. Most patients eat with others and eat less than four meals a day. The average dietary energy of patients with pulmonary tuberculosis in was 1242.6 ± 46.5 Kcal and 1084 ± 57.9 Kcal, respectively. 85.52% of patients did not eat enough food, 4.07% had enough, 10.41% consumed excess energy. The ratio of energy-generating substances in the diet (Carbohydrate:Protein:Lipid) was on average 54:18:28 for males and 55:16:32 for females. Most of the study population had diets that did not meet the experimental study in terms of micronutrient content. Specifically, more than 90% do not meet the requirements for magnesium, calcium, zinc, and vitamin D. The water-soluble and fat-soluble vitamins respond poorly, only about 30–40%. Selenium is the mineral with the best response rate, above 70%. Our findings revealed that the majority of the study subjects had poor nutritional status, as evidenced by diets lacking in essential micronutrients.

## 1. Introduction

Pulmonary tuberculosis is an infectious disease caused by *Mycobacterium tuberculosis* bacteria that primarily affects the lungs of infected individuals. The bacteria are mainly transmitted through the respiratory tract via inhalation of airborne particles containing tuberculosis bacteria [1]. Despite medical advancements, tuberculosis remains a significant public health issue with high morbidity and mortality rates, particularly in developing and underdeveloped countries. According to the Global Tuberculosis Report 2017, Vietnam ranks 16^th^ out of the 30 countries with the highest tuberculosis burden in the world with an estimated 130,000 new cases and 14,000 deaths annually [1]. In which, pulmonary tuberculosis is the most common form, accounting for about 80–85% of all TB cases and is the main source of infection in the community [2].

Studies have shown that malnutrition is an important factor in increasing the risk of TB in addition to many other factors affecting TB status [3]. Normally, people who carry TB bacteria will have no symptoms because the immune system controls the bacteria. However, malnourished individuals are more likely to develop active tuberculosis because of impaired immune function, including macrophages and T lymphocytes [4],[5]. On the contrary, TB also causes malnutrition, like other infections, TB consumes more energy for metabolism and leads to weight loss [6]. Furthermore, reduced food intake in TB patients due to anorexia and gastrointestinal symptoms also contributes to malnutrition [7]. A few previous studies investigating the nutritional status of patients with pulmonary tuberculosis showed a relatively low mean body mass index (approximately 17 kg/m^2^) [8]–[10]. Results of previous studies on patients with pulmonary tuberculosis showed that the rate of malnutrition according to body mass index (BMI) was 48.4%; according to the subjective global assessment method (SGA) was 56.1% [11],[12]. The study by Lal M Gurung showed that more than one third of the patients in the study were malnourished (as measured by BMI) [13].

Evaluation of dietary nutrient intake is very important in the nutritional management of patients with pulmonary tuberculosis. Insufficient calories and protein intake can interfere with the body's protective function of the immune system [14]. In addition, vitamins and minerals both play an important role in immunity [15],[16], and a lack of one or more of these nutrients reduces the body's resistance to infectious agents [17]. Research on nutritional intake of Chinese tuberculosis patients in 2019 by Zhewen Ren showed that the average dietary energy of patients was significantly lower than the recommended, to 87.4% male patients and 59.9% female patients not consume energy; most patients do not have enough protein requirements (90.8% men and 58.4% women) [7].

Therefore, research on nutritional status and dietary intake of patients with pulmonary tuberculosis is the basis to help develop interventions in clinical nutrition practice and treatment during hospital stay improve the patient's quality of life. However, in Vietnam, there are very few reports on the prevalence severity of malnutrition as well as the response to daily diet of patients with pulmonary tuberculosis. The Department of Respiratory Tuberculosis of the National Lung Hospital is a leading specialty in Vietnam specializing in the treatment of tuberculosis, including pulmonary tuberculosis. Every year, the facility receives and treats many cases from many localities across the country. With the desire to assess nutritional status and learn about the dietary intake of patients with pulmonary tuberculosis objectively and comprehensively in order to promptly make recommendations to improve the effectiveness of treatment, we carried out this study with the aim nutritional status and dietary intake before hospital admission of pulmonary tuberculosis patients at the Department of Respiratory Tuberculosis, National Lung Hospital, Vietnam.

## 2. Methods

### 2.1. Research subjects

The study was conducted on a total of 221 patients with tuberculosis who were admitted to the Respiratory Tuberculosis Department, National Lung Hospital from July 2019 to May 2020.

*Selection criteria:* Patients aged 18 years or older, diagnosed with pulmonary tuberculosis and hospitalized within 0–48 hours.

*Exclusion criteria:* The patient was incapable of listening, understanding and answering; patients with kyphosis scoliosis, pregnant women or those having serious complications emergency care.

### 2.2. Study design

This is a cross-sectional descriptive study.

*Sample size:*

Apply the formula for calculating sample size to a population proportion:

$$n=Z_{1-\alpha /2}^{2}\frac{p\left(1-p\right)}{{\left(\varepsilon p\right)}^{2}}$$

In which:

n: minimum sample size, p: rate of malnutrition of patients in the previous study, ε: relative deviation (choose ε = 0.2), Z: confidence coefficient in α (with α = 0.05 then Z_1-α/2_ = 1.96).

With p = 0.484 being the rate of malnutrition according to BMI, we have n = 102 [11]

With p = 0.307 being the rate of malnutrition according to MUAC, we have n = 216 [18]

With p = 0.561 being the rate of malnutrition according to SGA, we have n = 75 [12]

With p = 0.65 being the rate of malnutrition according to albumin, we have n = 51 [11]

For the sample size to satisfy all evaluation methods, we have a minimum sample size of 216. Actually collected 221 subjects.

*Sampling method:*

Convenience sampling was completed until 221 patients with a confirmed diagnosis of pulmonary tuberculosis were eligible to participate in the study at the Respiratory Tuberculosis Department of the National Lung Hospital.

### 2.3. Variables

To assess the nutritional status of the patient, the questionnaire included: anthropometric indices, the Subjective Global Assessment (SGA) questionnaire, and arm circumference measurement (MUAC). Data were collected by direct interview method according to a prepared questionnaire.

Measurement of the anthropometric index is described in [19],[20].

The anthropometric measurements were taken using a digital scale with precision of ± 0.1 kg. The scale was placed on a flat surface, and the subjects wore thin clothing and did not carry any devices such as watches or phones. The unit of measurement was kg, and the results were recorded with one decimal place (e.g., 45.5 kg).

The height of the subjects was measured using a wooden stadiometer with a 0.1 cm graduation. During the measurement, the subjects removed their shoes, hair accessories, and stood with their back against the stadiometer, ensuring that 9 points of contact were made: 2 heels, 2 calf muscles, 2 buttocks, 2 shoulders, occiput, and the eyes looking straight ahead. The vertex of the head was aligned perpendicular to the stadiometer using a sliding headpiece. The height measurement was recorded to one decimal place with the unit of cm.

Body mass index (BMI) was calculated as weight in kilograms divided by the square of height in meters, and the nutritional status of adult patients was determined according to criteria for such as normal weight: 18.5–22.9 kg/m^2^, overweight: 23–24.9 kg/m^2^, and obese ≥ 25 kg/m^2^ [21].

Criteria for assessing nutritional status according to nutrition institutes by MUAC index (Female: malnourished when MUAC < 23 cm; Male: malnourished when MUAC < 24 cm). The most commonly used measurement ring is the non-dominant arm measurement, the natural hanging position. Use a soft, non-elastic ruler with precision of ± 0.1 cm. The measuring loop passes through the midpoint of the arm from the sacral process to the superior condylar process of the humerus [22].

The Subjective Global Assessment (SGA) [23] was developed in 1980 and is the only tool recommended by the American Society for Parenteral and Enteral Nutrition (ASPEN) to assess the nutritional status of hospitalized patients within 48 hours of admission. The SGA is a non-invasive, reliable, specific, and cost-effective tool for patients under 65 years old. The assessment technique is based on changes in weight, changes in food intake, gastrointestinal symptoms, changes in functional mobility, underlying diseases, the impact of metabolic stress, and clinical signs of malnutrition (subcutaneous fat loss, edema, and muscle wasting), which are scored from 0–2 points. The classification includes: Well-nourished (SGA-A), At-risk for malnutrition (SGA-B), and Malnourished (SGA-C).

To assess typical dietary intake, 221 participants completed 24-hour dietary recall,a method that includes recalling, describing, and accurately quantifying food and beverage intake consumed during the 24-hour period in the day before the interview (from the first meal or drink in the morning to the last meal or drink at night) [22].

Nutritional status assessment based on Albumin is described in [24]: is a protein synthesized by the liver that is correlated with chronic malnutrition and disease prognosis. The advantage of this method is that it is easy to perform and inexpensive, it is widely used for regular nutritional status assessments in hospitals and long-term treatments. The disadvantage of Albumin is its long half-life (21 days), and low levels of Albumin reflect prolonged malnutrition prior to the test. Classification: Severe malnutrition (albumin: < 21g/l); moderate malnutrition (albumin: 21- < 28 g/l); mild malnutrition (albumin: 28- < 35 g/l); normal nutritional status (albumin: 35–50 g/l).

**Table:** 

**Criterion for evaluation**	**Classification**		
	SGA - A	SGA - B	SGA - C
Weight loss within 6 months	N/R (Not Recorded)	5−10%	>10%
Change in dietary intake	N/R (Not Recorded)	Concentrated porridge, high-energy fluid	Low-energy liquid
Gastrointestinal symptoms	N/R (Not Recorded)	Loss of appetite	Nausea, vomiting
Reduced mobility	Normal	Decrease	Bedridden
Metabolic stress.	N/R (Not Recorded)	Normal	Severe
Clinical examination	Normal	Reducing subcutaneous fat and muscle mass	Presence of edema/ascites

### 2.4. Statistical analysis

Data was checked and converted before being entered into the computer by using Kobotoolbox software. This research has used statistical tests to analyze: frequency, rate mean for descriptive statistics; Chi-square test to compare 2 ratios. The analysis was performed by STATA 15.2 software. Diet of the study subjects was converted using the Vietnamese food composition table.

## 3. Results

**Table 1.** General information of research subjects.

Characteristics		n	%
Gender	Male	151	68.3
	Female	70	31.7
Age groups (year)	18–65	168	76.0
	>65	53	24.0
	Average ± SD = 51.3 ± 17.1; min = 19; max = 89
Nation	Kinh	210	95.5
	Other Ethnicities	11	4.5
Accommodation	Urban	120	54.3
	Rural	101	45.7
Occupation	Farmers	68	30.8
	Workers, Officials	43	19.5
	Housewife, Retirees, Freelance-workers	104	47.0
	Students	6	2.7
Education level	Illiteracy	2	0.9
	Elementary-Middle school	99	44.8
	High school	81	36.7
	College, university	38	17.2
	Post-Graduate	1	0.4

According to Table 1, the proportion of male patients was higher than that of female patients; average age was 51.3 ± 17.1, with the majority falling in the group of 18–65 years old. The majority of the subjects were Kinh people (95.5%) and lived in rural areas (45.7%); TB patients were predominantly retirees, freelance-workers (47%), with only 2.7% being students. Most of the patients had lower secondary school education (44.8%), very few were illiterate (0.9%).

**Table 2.** The nutritional status of pulmonary tuberculosis patients was assessed using BMI, MUAC, SGA, and Albumin.

Nutritional status		n	%
BMI (n = 199)	Malnutrition grade III (<16 kg/m^2^)	25	12.7
	Malnutrition grade II (16−17 kg/m^2^)	15	7.5
	Malnutrition grade I (17−8.5 kg/m^2^)	51	25.6
	Normal (18.5−22.9 kg/m^2^)	88	44.2
	Overweight/obese (≥23 kg/m^2^)	20	10.0
MUAC (n = 221)	Malnutrition	133	60.2
	Normal	88	39.8
SGA (n = 221)	SGA-A	93	42.1
	SGA-B	90	40.7
	SGA-C	38	17.2
Albumin (n = 156)	Normal	78	50
	Mild malnutrition (28- < 35 g/l)	45	28.9
	Moderately malnourished (21- < 28 g/l)	28	17.9
	Severe malnutrition (<21 g/l)	5	3.2

Table 2 presents the nutritional status of pulmonary tuberculosis patients upon hospital admission. Based on BMI, 45.8% of patients were classified as malnourished, 44.2% were normal weight, and 10.0% were overweight or obese. According to MUAC, 60.2% of patients were malnourished, while 39.8% were normal. SGA classification showed that 57.9% of patients were at risk of malnutrition, with 40.7% at moderate risk and 17.2% at severe risk. Based on Albumin levels, 50% of patients were malnourished, with rates of mild, moderate, and severe malnutrition at 28.9%, 17.9%, and 3.2%, respectively.

**Table 3.** The Relationship of nutritional status between classification based on BMI, SGA and MUAC.

Characteristics	Classification according to BMI n (%)			*p*
	BMI < 18.5	18.5 ≤ BMI< 23	BMI ≥ 23	
Classification according to SGA			
Normal	23 (25.0)	57 (65.5)	11 (55.0)	*0.000^a^*
Risk of malnutrition	69 (75.0)	30 (34.5)	9 (45.0)	
Classification according to MUAC		
Normal	11 (12.0)	54 (62.1)	17 (85.0)	*0.000^a^*
Malnutrition	81 (88.0)	33 (37.9)	3 (15.0)	

*Note: ^a^ Chi-square test.

Table 3 demonstrated that, among individuals with BMI < 18.5, the majority of cases of malnutrition were identified using both SGA and MUAC classifications (with 75.0% and 88.0%, respectively). In contrast, in the two other BMI groups, the rate of malnutrition according to SGA was 19.6%; malnutrition according to MUAC was 18.1%. Comparing the nutritional status based on BMI and SGA classifications revealed a statistically significant difference (P < 0.01). Similarly, there was a statistically significant difference between nutritional status according to BMI and MUAC classifications (with *p < 0.01*).

**Table 4.** Nutritional value of 24-hour diet of study subjects.

Characteristics (n = 221)			Average ± SD	95% CI
Energy (Kcal)		Male	1242.6 ± 46.5	1150.6–1334.5
		Female	1084 ± 57.9	968.9–1200.1
Carbohydrates	The ratio of Carbohydrate (%)	Male	53.8 ± 1.1	51.7–55.9
		Female	54.5 ± 1.2	52.0–56.9
Protein	The ratio of Protein (%)	Male	17.7 ± 0.7	16.4–19.0
		Female	16.3 ± 0.5	15.3–17.4
	Total Protein (g)	Male	51.4 ± 2.2	47.0–55.8
		Female	43.2 ± 2.5	38.2–48.1
	Animal protein/total (%)	Male	56.4 ± 1.7	53.1–59.7
		Female	53.6 ± 2.5	48.6–58.6
Lipid	The ratio of Lipid (%)	Male	28.1 ± 0.8	26.6–29.5
		Female	32 ± 2.8	26.4–37.6
	Total Lipid (g)	Male	39.4 ± 1.9	35.6–43.2
		Female	36.2 ± 2.6	30.9–41.4
	Plant lipid/total (%)	Male	58.8 ± 1.8	55.3–62.3
		Female	63.6 ± 2.4	58.8–68.3

The nutritional values of the 24-hour diet of the study subjects were presented in Table 4. The average dietary energy of patients with pulmonary tuberculosis in male and female was 1242.6 ± 46.5Kcal and 1084 ± 57.9kcal, respectively. The ratio of energy-generating substances in the diet (Carbohydrate:Protein:Lipid) was on average 54:18:28 for males and 55:16:32 for females. The average animal protein/total protein ratio, which met the recommended requirement (>30%). The mean vegetable/total lipid ratio for was 63.6%, within the recommended range (>60%), while that for 58.8%, lower than the recommended range.

Table 5 presents the results of micronutrient content in the 24-hour diet of the study subjects. The majority of patients had diets that did not meet the recommended requirement for micronutrients. Specifically, more than 90% did not meet the requirements for magnesium, calcium, zinc, vitamin D. However, selenium was the mineral with the best response rate, at over 70%. The rate of meeting the recommended requirement for vitamins in the 24-hour diet was low, approximately 30–40%.

**Table 5.** Vitamin and mineral content through analysis of 24-hour diets of study subjects.

	Average ± SD	95% CI	Unsatisfactory n (%)	
			Male	Female
***Minerals***
Mg(mg)	136.5 ± 90.2	124.5–148.5	145(96.0)	64(91.4)
Ca(mg)	412.7 ± 281.5	375.4–450.1	138(91.4)	64(91.4)
P(mg)	608.4 ± 320.9	565.9–651.0	94(62.3)	54(77.1)
Ca/P(mg)	0.72 ± 0.47	0.65–0.78	122(80.8)	55(78.6)
Fe(mg)	9.13 ± 5.92	8.34–9.91	90(59.6)	67(95.7)
Cu(µg)	855.1 ± 486.7	790.5–919.6	86(60.0)	41(58.6)
Zn(mg)	7.18 ± 5.93	6.39–7.96	143(94.7)	66(94.3)
Se(µg)	67.4 ± 39.9	62.1–72.7	35(23.2)	10(14.3)
***Vitamins***
Vitamin A(µg)	274.6 ± 343.4	229–320.1	142(94.0)	62(88.6)
Vitamin D(µg)	3.46 ± 6.59	2.58–4.3	141(93.4)	65(92.9)
Vitamin E(mg)	4.76 ± 3.77	4.3–5.3	117(77.5)	48(68.6)
Vitamin K(µg)	276.7 ± 409.9	222.4–331	98(64.9)	43(61.4)
Vitamin B1(mg)	1.01 ± 0.57	0.94–1.09	97(64.2)	38(54.3)
Vitamin B2(mg)	0.74 ± 0.52	0.67–0.81	135(89.4)	60(85.7)
Vitamin C(mg)	124.2 ± 107.7	109.9–138.4	82(54.3)	29(41.4)

## 4. Discussion

The study was conducted on 221 patients who were diagnosed with pulmonary tuberculosis at the National Lung Hospital in 2019–2020 during the period from July 2019 to May 2020.

In our study, the majority of the study participants were male, which is consistent with the findings of previous domestic and foreign studies such as the study of Le TT in 2018 [11], the study on the nutritional status of pulmonary tuberculosis patients in Nepal [13] and a study on adults with pulmonary tuberculosis in India had a similar ratio of male to female patients with our results [8]. Subjects in our study had an average age of 51.3 ± 17.1 and more than three-quarters of the study subjects were in the 18–65 age group. This result was similar to the results of some previous studies [25]–[27]. More than 95% of the subjects in the study were from the Kinh ethnic group, because the Kinh people for the majority of Vietnam's population. The rate of TB patients in rural areas was the highest, but the rate in big cities was also not small. This rate can be explained because Vietnam is a developing country with most of its territory in rural areas. However, the rate of TB patients in big cities was also relatively high due to the population living and working there. Most of the study subjects had completed the secondary and high school, which is similar to the study of Le TT [11].

BMI is a widely used nutritional assessment index for evaluating the nutritional status of individuals. Measuring BMI within the first 48 hours of admission to the hospital can provide medical staff with timely information on the patient's nutritional status for the best care and intervention plans. In our study, 199 patients were eligible for BMI assessment and the average BMI of the study subjects was 19.1 ± 2.8 kg/m^2^. Our results were higher than the results of some previous studies in Vietnam [11],[28], but lower than the results of the studies on pulmonary TB patients in Nepal (20.99 kg/m^2^) [13] and Brazil (21 ± 3.9 kg/m^2^) [29]. These differences could be attributed to various factors such as race, time of study and average value of BMI influenced by the distribution of values in the observed sample. The rate of malnutrition among the study subjects was quite high, this result was similar to some previous studies in Vietnam [11],[25] and higher than some studies in Nepal and Brazil [13],[30], the result of our study was much lower than the study in India [8]. These differences could also be due to variations in race, socioeconomic conditions, and sample size. Anthropometry is an essential index for the initial assessment of nutritional status because it is a simple, widely applicable, inexpensive, and non-invasive technique requiring only easy-to-carry equipment. However, there have been studies that have shown a lack of sensitivity of BMI in assessing nutritional status [31],[32], whereby MUAC measurement has been proposed as an alternative anthropometric parameter for nutritional diagnosis [33]. In this study, the mean MUAC value of the subjects was 22.4 ± 2.9 cm which was smaller than the reference value for both men and women. The rate of malnourished subjects according to MUAC was quite high. Our results were different from the study in patients with pulmonary tuberculosis in Brazil [30]. There was a difference due to many factors, the most basic of which was the choice of different reference values. The nutritional status assessed by BMI was different from that assessed by MUAC (the difference was statistically significant), and this was a difference due to the different evaluation criteria of each method. Thereby, we believe that it is necessary to use a combination of many methods in assessing the nutritional status of patients to avoid missing cases that need attention intervention.

In addition to the anthropometric nutritional status assessment methods, there is also a comprehensive assessment of nutritional status according to SGA developed by Destky [23]. This method is concerned with the weight change, eating capacity, gastrointestinal symptoms, ability to live, diseases, risks and potential factors for malnutrition. SGA is a subjective method for measuring nutritional status, however it is quick and easy to perform and is also non-invasive. SGA was shown to be a useful tool for nutritional assessment of patients with pulmonary tuberculosis, this assessment should be performed at the time of admission to capture the patient's clear weight loss status and timely intervention before entering treatment [34]. In our study, subjects were at risk of malnutrition according to SGA. This result was similar to some previous studies [12],[34]. There was a difference in the prevalence of malnutrition as assessed by BMI and by SGA. Considering the relationship between nutritional status according to BMI and SGA, there were patients with BMI < 18.5, but not malnourished as assessed by SGA, there were patients with malnutrition as assessed by SGA but BMI ≥ 18.5 kg/m^2^. This was a difference due to the different evaluation criteria in each method. For example, in the method of using BMI, if the patient has edema or weight loss in the overweight person, if we do not pay attention, it is easy to determine the incorrect result. Therefore, it is necessary to have a general assessment by many methods to have a more accurate assessment in health care, especially in high-risk subjects such as tuberculosis patients.

Albumin is a commonly used parameter to assess visceral protein reserves [35]. A decline in serum albumin levels is associated with increased morbidity and mortality in hospitalized patients [36]. In this study, the ratio of malnourished patients according to based on Albumin was lower than that of the author Le TT [11], and higher than the study on pulmonary tuberculosis patients in Brazil [30]. The differences may be due to the different sizes and designs of the studies and the factors that affect albumin in different patients.

To clarify the role of diet in the prevention and treatment of pulmonary tuberculosis, we investigated the 24-hour diet of all study subjects in the day before hospital admission to objectively study subjects. Research results show that the average energy consumption was much lower than the average energy consumption of Vietnamese people according to the results of the 2020 Nutrition Census of the Institute of Nutrition [37]. This difference is due to differences in disease status, severity and impact of drugs, inflammatory mechanisms, etc. affecting food consumption. Our results were also much lower than some previous studies on pulmonary tuberculosis patients [7],[8],[38]. The difference may be due to economic conditions, socio-cultural characteristics and eating habits of different countries. Vitamins and minerals both play an important role in immunity, a lack of one or more of these micronutrients will lower resistance to any infection [6],[15]–[17],[39]. Through analyzing the 24-hour diet of the study subjects, we found that the response rate of micronutrients is very poor compared to the recommended needs for Vietnamese people [40].

This study is one of the very few studies investigating the nutritional status and dietary intake of pulmonary tuberculosis patients, which is a relatively new issue in Vietnam. Therefore, more research is needed on this issue in the future to have more complete data.

In addition, due to limited resources, we used in the 24-hour dietary recall methods to survey but we only performed the survey in one day, which may cause certain errors, so we also try to adhere to the method and also achieved some similar results with previous studies.

## 5. Conclusions

In this study, we utilized various methods to assess the nutritional status of 221 patients diagnosed with pulmonary tuberculosis. Our findings revealed that the majority of the study subjects had poor nutritional status, as evidenced by diets lacking in essential micronutrients. These results underscore the need for public health initiatives aimed at enhancing the quality of care and nutrition programs for tuberculosis patients. These initiatives should include screening and nutritional assessments, particularly the subjective overall assessment method SGA, upon hospital admission to inform patient care. Furthermore, a comprehensive nutritional intervention plan should be developed for patients, encompassing counseling, communication, answering questions, and guidance on diet and exercise activities tailored to individual patients.

## References

[1] World Health Organization (‎2018). Global tuberculosis report 2018.

[2] Nguyen VC (2006). Tuberculosis Pathology.

[3] Feleke BE, Feleke TE, Biadglegne F (2019). Nutritional status of tuberculosis patients, a comparative cross-sectional study. BMC Pulm Med.

[4] Chai Q, Zhang Y, Liu CH (2018). Mycobacterium tuberculosis: An Adaptable Pathogen Associated With Multiple Human Diseases. Front Cell Infect Microbiol.

[5] Macallan D (2009). Infection and malnutrition. Med.

[6] Gupta KB, Gupta R, Atreja A (2009). Tuberculosis and nutrition. Lung India.

[7] Ren Z, Zhao F, Chen H (2019). Nutritional intakes and associated factors among tuberculosis patients: a cross-sectional study in China. BMC Infect Dis.

[8] Bhargava A, Chatterjee M, Jain Y (2013). Nutritional Status of Adult Patients with Pulmonary Tuberculosis in Rural Central India and Its Association with Mortality. PLoS One.

[9] Musuenge BB, Poda GG, Chen PC (2020). Nutritional Status of Patients with Tuberculosis and Associated Factors in the Health Centre Region of Burkina Faso. Nutrients.

[10] Ko Y, Kim C, Park YB (2020). Changes in Nutritional Status in Pulmonary Tuberculosis: Longitudinal Changes in BMI According to Acid-Fast Bacilli Smear Positivity. J Clin Med.

[11] Le TT, Le VH, Nguyen TH (2019). Characteristics tuberculosis of the patients who hospitalized at the Respiratory Tuberculosis Department, National Lung Hospital in 2018. J Vietnam Med.

[12] Le TT, Le VH, Nguyen TLH (2019). Nutritional status using subjective global assessment method and some risk factors in patients with pulmonary at the Respiratory Tuberculosis Department, National Lung Hospital in 2018. J Vietnam Med.

[13] Gurung LM, Bhatt LD, Karmacharya I (2018). Dietary Practice and Nutritional Status of Tuberculosis Patients in Pokhara: A Cross Sectional Study. Front Nutr.

[14] WHO (2013). Guideline: Nutritional care and support for patients with tuberculosis.

[15] Chandrasekaran P, Saravanan N, Bethunaickan R (2017). Malnutrition: Modulator of Immune Responses in Tuberculosis. Front Immunol.

[16] Wagnew F, Alene KA, Eshetie S (2022). Effects of zinc and vitamin A supplementation on prognostic markers and treatment outcomes of adults with pulmonary tuberculosis: a systematic review and meta-analysis. BMJ Global Health.

[17] Gombart AF, Pierre A, Maggini S (2020). A Review of Micronutrients and the Immune System–Working in Harmony to Reduce the Risk of Infection. Nutrients.

[18] Pham TH (2019). Nutritional status and some related factors in Graves patients at the National Hospital of Endocrinology in 2019. Graduation thesis, Hanoi Medical University.

[19] Phạm DT, Hà DK (2006). Guidelines for nutritional practice in the community.

[20] Ha HK (1997). Nutritional epidemiological methods.

[21] WHO Expert Consultation (2004). Appropriate body-mass index for Asian populations and its implications for policy and intervention strategies. Lancet.

[22] National Institute of Nutrition (2015). Assess nutritional status and monitor growth. J Nutr Food.

[23] Detsky AS, McLaughlin JR, Baker JP (1987). What is subjective global assessment of nutritional status?. JPEN J Parenter Enteral Nutr.

[24] National Cancer Institute (2017). 24-hour Dietary Recall (24HR) At a Glance.

[25] Seltzer MH, Bastidas JA, Cooper DM (1979). Instant nutritional assessment. JPEN J Parenter Enteral Nutr.

[26] Duong QT (2016). Relationship between BMI with some clinical and subclinical characteristics of pulmonary tuberculosis and the change of BMI after 1 month of treatment. J Pharm - Hue Pharm Univers.

[27] Owolabi OA, Jallowa AO, Jallowa M (2020). Delay in the diagnosis of pulmonary tuberculosis in The Gambia, West Africa: A cross-sectional study. Inter J Infect Disea.

[28] Diriba K, Churiso G (2022). The prevalence of Mycobacterium tuberculosis using Gene Xpert among tuberculosis suspected patients in Gedeo Zone, Southern Ethiopia. Euro J Med Resear.

[29] Hoang KTA, Tran TVA, Pham TD (2018). Nutritional status in tuberculosis patients treated at Thai Binh Lung Hospital in 2017. J Nutr Food.

[30] Bacelo AC, Ramalho A, Brasil PE (2015). Nutritional Supplementation Is a Necessary Complement to Dietary Counseling among Tuberculosis and Tuberculosis-HIV Patients. PLoS One.

[31] Oliveira CMC, Kubrusly M, Mota RS (2010). Malnutrition in chronic kidney failure: what is the best diagnostic method to assess?. J Bras Nefrol.

[32] Al Moamary MS, Alorainy H, AL-Hajjaj MS (2014). Pulmonary rehabilitation: A regional perspective evidenced-based review. Ann Thorac Med.

[33] Chakraborty R, Bose K, Koziel S (2011). Use of mid-upper arm circumference in determining undernutrition and illness in rural adult Oraon men of Gumla District, Jharkhand, India. Rural Remote Health.

[34] Lin HS, Lin MS, Chi CC (2021). Nutrition Assessment and Adverse Outcomes in Hospitalized Patients with Tuberculosis. J Clin Med.

[35] Sergi G, Coin A, Enzi G (2006). Role of visceral proteins in detecting malnutrition in the elderly. Eur J Clin Nutr.

[36] Akirov A, Masri-Iraqi H, Atamna A (2017). Low Albumin Levels Are Associated with Mortality Risk in Hospitalized Patients. Am J Med.

[37] National Institute of Nutrition, Vietnam (2021). Press release Conference announcing the results of the 2019-2020 Nutrition Census.

[38] Macallan DC (1999). Malnutrition in tuberculosis. Diagn Microbiol Infect Dis.

[39] Huang Z, Liu Y, Qi G (2018). Role of Vitamin A in the Immune System. J Clin Med.

[40] Vietnam Ministry of Health, Institute of Nutrition (2016). Recommended nutritional needs for Vietnamese people.

